# History and growth of radiation oncology in Indonesia

**DOI:** 10.2349/biij.4.3.e42

**Published:** 2008-07-01

**Authors:** SA Gondhowiardjo, GB Prajogi, SM Sekarutami

**Affiliations:** Department of Radiotherapy, Cipto Mangunkusumo General Hospital, Jakarta, Indonesia

**Keywords:** Radiotherapy, Indonesia, profile

## Abstract

In order to assess its progress, and to obtain a snapshot of the current situation, the Indonesian Radiation Oncology Society has routinely conducted annual surveys since 2004 to assess the current condition of resources for equipment and staffing in all radiation oncology centres in the country. Based on these routine surveys, the society has made recommendations to the government about providing cancer patients with better and more affordable access to radiotherapy services.

Questionnaires in hard and soft copy forms were distributed annually to Indonesian Radiation Oncology Society members, and all 22 centres (19 in 2004) responded by sending back the completed questionnaires. The obtained data was compared with results of the first survey in 2004.

In 2008, there were 22 radiotherapy centres in Indonesia, 18 Linear Accelerators and 17 cobalt units. There were 270 radiation oncology professionals, including 41 radiation oncologists, 38 medical physicists, 6 dosimetrists, 125 radiation therapy technologists, and 60 nurses. In addition, there were 17 residents and trainees participating in the Radiation Oncology training program.

A lot of progress had been made in the four-year period from 2004 to 2008. Indonesia has seen the establishment of 4 new centres, which indicates a 50% increase in the number of available treatment units, and a 29% increase in the number of human resources. These achievements were possible because more attention has been placed on cancer care issues in Indonesia, a great success due to the advocacy efforts of the Society. However, numerous issues still need urgent attention from all the stakeholders.

## INTRODUCTION

### History of Radiotherapy Services in Indonesia

It has been more than 80 years since the first radiotherapy treatment was carried out in Indonesia. A lot has been accomplished in this rapidly evolving field, which saw, among its highlights, the establishment of the Indonesian Radiation Oncology Society in July 2000.

The history of radiation oncology in Indonesia is closely related to the development of radiology services in the country. It was initiated in 1927 by B. Van der Plaats, a Dutch radiologist, to complement the existing radiology services in the Central General Hospital (now Cipto Mangunkusumo General Hospital). The only available equipment at that time was a conventional X-ray therapy unit, which is mainly used to treat superficial skin lesions [1].

After the country’s independence in 1945, Prof W.Z. Johannes, the first Indonesian radiologist, continued the development of radiotherapy services in Indonesia. In the following years, additional superficial and deep X-ray therapy units were installed in the central general hospital to accommodate the growing need for radiotherapy service in the hospital, and several Indonesian doctors were sent to foreign institutions for training in radiology and radiotherapy [1]. Among these early radiation oncologists were Prof. GA Siwabessy, who succeeded Prof W.Z. Johannes after his death in 1952 [2].

A serious step towards better radiotherapy services was made with the installation of one Cobalt-60 teletherapy unit in 1958. The Cobalt teletherapy unit was initially placed several kilometers from the hospital, but was then moved in 1969 to the newly constructed radiotherapy facility inside the hospital, together with a Cesium-137 teletherapy unit which was installed in 1964 [1, 2]. Intracavitary radium brachytherapy has been carried out since 1958 in the Department of Gynecology, and remained there until 1982, after which it was carried out in the radiotherapy installation [2].

Since 1960, several other radiotherapy centres were established in other areas in Indonesia. These early centres included Surabaya, Jogjakarta, Semarang and Bandung, and were mostly equipped with kilovoltage and cesium teletherapy units [2].

In the period of 1980-1990, the Indonesian government established additional centres, particularly outside the island of Java, to provide better access to radiotherapy for patients with cancer. These centres were equipped with telecobalt units [2]. The country’s first linear accelerators, simulator and treatment planning system were installed in Cipto Mangunkusumo General Hospital in 1982 [1, 2].

A few more radiotherapy centres were established in the period of 1990-2000, in the Dharmais Cancer Hospital (1993), Persahabatan Hospital (1996) and in several other regional hospitals [2].

### The Indonesian Radiation Oncology Society

During its 70 years of existence in Indonesia, radiation oncology was organised as part of the discipline of radiology. The establishment of the Indonesian Radiation Oncology Society (IROS) in July 2000 set the cornerstone for the emergence of an independent discipline of radiation oncology.

As a society of radiation oncology professionals, with the vision of improving the quality of life of cancer patients in Indonesia, the Society had stated its missions as: (1) protecting the interests of public and patients; (2) guiding doctors; and (3) empowering the radiation oncology profession [3]. The Society actively advocated the advancement of Radiation Oncology in Indonesia, covering the quality assurance program as well as the technology issues in radiation oncology. To achieve its mission objectives, the Indonesian Radiation Oncology Society embraced various stakeholders in the field of radiation oncology services in Indonesia, including the Ministry of Health, Nuclear Energy Control Board (BAPETEN), National Atomic Energy Agency (BATAN), the Indonesian Medical Association, and Oncology Societies, as well as the pharmaceuticals and equipment companies.

The Society also cooperated with the Government Insurance Company concerning the structure of National Radiotherapy Services Tariff (insurance reimbursement system), to solve the issue of limited budget allocation from the government for the maintenance and replacement of equipments. Adopting the new tariff structure, each centre could independently finance their operations, including periodic replacement of old equipment and upgrading of their services, while maintaining accessible and affordable radiotherapy services to their patients.

Based on published IAEA standards, the Society issued the Professional Standards for Radiation Therapy Service, Standards of Competencies as a Radiation Oncologist, Standards of Training Program, and divided the Radiation Oncology Services into three categories: the primary, secondary as well as tertiary levels, each with its own minimum requirements of human resources, facilities including dosimeters as well as procedure of self assessment.

In late 2007, the Indonesian Medical Association and Indonesian College of Medicine approved the Indonesian College of Radiation Oncology under the IROS, and radiation oncology became an independent discipline.

### Regulatory Boards

In 1954, a National Committee for the Investigation of Radioactivity was formed to investigate the possibility of a radioactive fall-out in Indonesian territory due to nuclear weapon tests in the Pacific during the Cold War. However, this committee was short-lived. Adopting the IAEA principle, “Atoms for Peace”, the National Council for Atomic Energy was later established on December 5th 1958, and in 1964 it became the National Atomic Energy Agency [4].

It was only after 1997 that the government decided to have separate agencies for the research and development of atomic energy (BATAN) and for the regulation of atomic energy (Nuclear Energy Control Board, NCEB / BAPETEN) [5].

All radiation oncology centres in Indonesia are supervised by the Nuclear Energy Control Board as the regulatory board for application of atomic energy. The board reports directly to the President of the Republic of Indonesia, and is responsible for supervision of the use of atomic energy, formulation of regulations, and the issue of operating licenses which are re-evaluated over certain periods to assure safety for the public, personnel, and patients [5, 6].

To complement the existing physics quality assurance program that is done under the supervision of the Nuclear Energy Control Board, IROS is currently developing more comprehensive annual self-assessment surveys to enable thorough evaluation of the national radiotherapy services, including human resources, facility, and the delivery of radiation treatments.

### Growth of Radiation Oncology in Indonesia

Cancer care had not received much priority until recent years. A good national cancer control program was still unavailable, and various cancer control efforts carried out by a number of institutions were largely uncoordinated. Accurate data on cancer incidence was still unattainable due to administrative, financial and geographic constraints. The recently published results of a pathological-based cancer registry, which was initiated in 2002 by the Indonesian Association of Pathologists, still lacked adequate coverage, with only 16224 cases registered from 11 provinces [7]. A hospital-based cancer registry has recently been re-initiated by the Ministry of Health, but this registry is currently under development and no data is available as yet.

The lack of a national cancer control program was a problem for institutions and organisations of cancer care, including the Indonesian Radiation Oncology Society. The young society, in cooperation with various societies in oncology, advocated stakeholders including the Ministry of Health about the current situation of cancer care, radiotherapy needs and capacity, requirements for facilities and human resources, quality assurance and radiation safety issues, and the need for technological advancement of existing radiation therapy. This increased the awareness of stakeholders, which resulted in more attention and support given to areas of cancer care and research in the recent years.

## MATERIALS AND METHODS

In order to assess its progress, and to obtain a snapshot of the current situation, the Indonesian Radiation Oncology Society has routinely conducted annual surveys since 2004 to assess the current condition of resources for equipment and staffing in all radiation oncology centres in the country. Based on these routine surveys, the society has made recommendations to the government on how to provide cancer patients with better and more affordable access to radiotherapy services.

Questionnaires in hard and soft copy forms were distributed annually to Indonesian Radiation Oncology Society members, and all 22 centres (19 in 2004) responded by sending back the completed questionnaires. The obtained data was compared with results of the first survey in 2004.

## RESULTS

### Radiation Oncology Centres

In 2008, there were 22 radiotherapy centres, a 21% improvement over the 2004 data. At the moment, 17 of them are operational. There are 2 centres providing radiotherapy services in Sumatra, 13 centres in Java, 1 centre in Bali and Nusa Tenggara, 1 centre in Kalimantan, and 1 centre in Sulawesi and the Eastern part of Indonesia. An additional five centres are currently inactive, with three undergoing equipment replacement and two new centres under commission. On average, there is 1 centre per 10 million population.

### Equipment and Facilities

There were 17 Cobalt-60 teletherapy units and 18 linear accelerators, mostly in major cities. Six of these treatment units are currently under commission. Considering major island groups as regions, the ratio of treatment units per million regional population ranged from 0.088 in Kalimantan to 0.222 in Java; a factor of 2.5. The median workload for the treatment machines was 353 patients/treatment unit annually (range: 102-800).

Three-dimensional Treatment Planning System (3D-TPS) is available in 11 centres, many of which only started using 3D-Conformal technique within the past 4 years. CT Simulators were available in 5 centres, and simulators in all but 1 centre. At the moment, IMRT is performed only in Cipto Mangunkusumo General Hospital, as the only unit with such available facilities and capabilities.

In 2008, there were 8 centres actively performing brachytherapy. HDR afterloading technique was used in all centres performing brachytherapy, and intracavitary insertions for cervical cancer was the most common brachytherapy procedure, constituting 50-75% of all brachytherapy procedures in the centres.

### Human Resources

In 2008, there were 270 people working in radiation oncology, including 41 radiation oncologists, 38 medical physicists, 6 dosimetrists, 125 radiation therapy technologists, and 60 nurses. In addition, there were 17 residents and trainees participating in the radiation oncology training program.

In 2004, there were only 205 radiation oncology personnel, which included 39 radiation oncologists, 27 medical physicists, 2 dosimetrists, 87 radiation therapy technologists, and 52 nurses. During the same period, there were only 4 trainees in this discipline.

Compared with the 2004 data, there has been a 31% increase in the radiation oncology personnel. Six new radiation oncologists completed their training program between 2004 and 2008, but 4 senior radiation oncologists had retired, resulting in a net increase of just 5%. To solve the problem of the aging community of radiation oncologists, the Society has started a revised radiation oncology residency training to prevent shortage of human resources.

## DISCUSSION

### Discussion

All currently active radiotherapy centres in Indonesia are government-owned, situated in central or regional public hospitals. The total number of patients receiving radiotherapy in 2007 was 10,274 for a total population of approximately 200 million, or in the region of 50 patients treated per million population.

A lot of progress has been made in the four-year period between 2004 and 2008. Indonesia has seen the establishment of 4 new centres, indicating a 50% increase in the number of available treatment units, and a 29% increase in the number of human resources. These achievements were possible because more attention has been placed on cancer care issues in Indonesia, a great success owing to the advocacy efforts of the Society. However, numerous issues still need urgent attention from all the stakeholders.

As an accurate cancer registration system is still unavailable, an estimated cancer incidence of 1 per 1000 produced an estimate number of 200,000 cancer patients [8]. If half of these patients with cancer were expected to benefit from radiotherapy, it was estimated that based on the current capacity, only 10% of all cancer patients had immediate access to radiotherapy services. This situation was reflected by the long waiting list in most radiotherapy centres in Indonesia, which exceeded 6 months in several centres. The government’s effort to provide additional centres has been hindered by restrictions in funding and the shortage of human resources. With only 38 radiation oncologists available, not many new centres can be established, considering that all the active radiation oncologists have been working at full capacity.

The distribution of radiotherapy centres has also been problematic. More than half of all centres are located in the island of Java, home to 114 million people. Of these, 5 are located in Jakarta, the capital city and the only province in Indonesia that can supply more than 1 megavoltage units per million population. In practice, however, these 5 centres located in Jakarta function as referral centres for almost all other parts of Indonesia and also serve as a regional centre for West Java, thus becoming overwhelmed by the number of patients. Operational hours exceeding 10 hours per day are not uncommon in several centres.

Access to radiotherapy services remains difficult in several provinces in Indonesia. Patients in many provinces have to travel several hundred to thousand kilometers to the nearest centres, sometimes over the sea. Only 2 centres currently provide radiotherapy services to the eastern part of the Indonesian territory, a maritime area of many islands covering nearly half the total area of Indonesia, home to more than 18 million people.

A 2001 survey done by Tatsuzaki, *et al*. showed that radiotherapy services in Indonesia still lagged behind most countries in the Asia-Pacific region [9]. Indonesia currently has 0.17 megavoltage treatment units per million population, a 33% increase compared with the 2001 value of 0.12. Eight additional radiation oncologists have been recruited, producing a 10% increase from the 2001 value of 0.16 radiation oncologists per million population. However, even with these increases, Indonesia was still behind most countries in the 2001 survey.

Comparison with more recent data from other Asian countries might show an even more disappointing picture. A recent 2008 report by Yin, *et al*. from the Chinese Radiation Oncology Society showed a massive increase in the number of treatment units and human resources [10]. When compared with the 2001 survey, China had achieved a 40% increase in the number of centres, and an almost 50% increase in the number of megavoltage units, an increase that brought them beyond the standard of 1 centre per million population.

**Figure 1:**
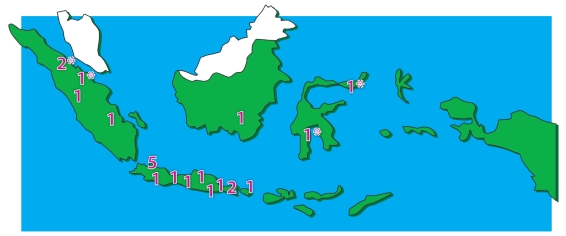
Distribution of radiotherapy centres in Indonesia. Numbers represent the number of radiotherapy centres in a city. *Centres that are currently inactive.

**Figure 2:**
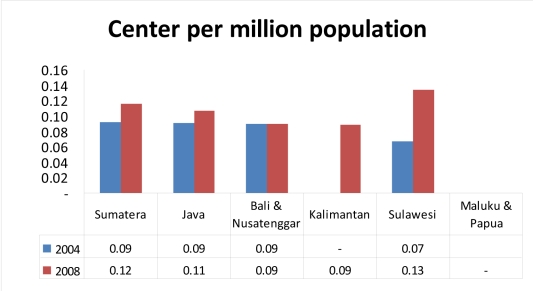
Centre to population ratio, divided into areas of major island groups. The 6 currently inactive centres (under commission) were also included in the calculation.

**Figure 3:**
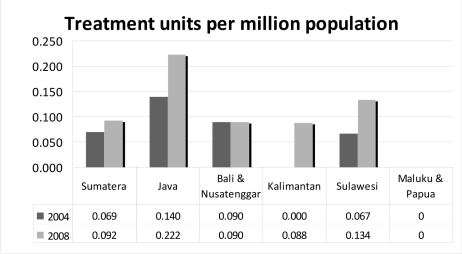
Treatment units per million population, divided by islands as regions.

**Figure 4:**
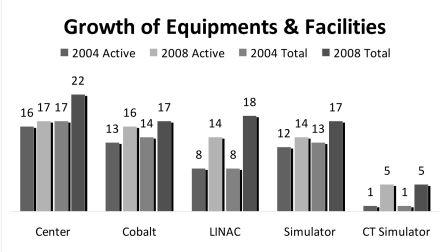
Growth of equipment and facilities, 2004-2008.

**Figure 5:**
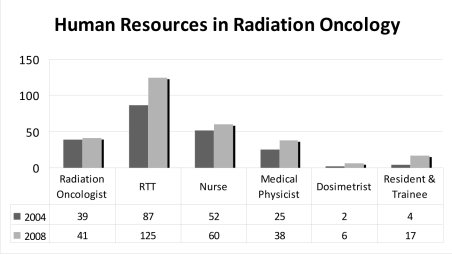
Growth of human resources, 2004-2008.

**Table 1:** Availability of equipment.

Equipment	n	%
LINAC/Co-60 + CT Simulator (IMRT)	1	4%
LINAC/Co-60 + CT Simulator (3D Conformal)	4	18%
LINAC/Co-60 +Simulator (3D Conformal)	11	50%
LINAC/Co-60 +Simulator (Simple Conventional)	5	24%
LINAC/Co-60	1	4%

**Table 2:** Radiotherapy services in Indonesian provinces.

**Province**	**Population (2000 Census)**	**Radiotherapy Centres**	**Cobalt**	**LINAC**	**MV units / million**
Nanggroe Aceh Darussalam	3,930,905	-	-	-	-
North Sumatra	11,649,655	2	-	1	0.086
West Sumatra	4,248,931	1	1	-	0.235
Riau	4,957,627	1	1	-	0.202
Jambi	2,413,846	-	-	-	-
South Sumatra	6,899,675	1	1	-	0.145
Bengkulu	1,567,432	-	-	-	-
Lampung	6,741,439	-	-	-	-
Bangka & Belitung	900,197	-	-	-	-
Jakarta	8,389,443	5	3	8	1.311
Banten	8,098,780	-	-	-	-
West Java	35,729,537	1	1	1	0.056
Central Java	31,228,940	3	4	1	0.160
Jogjakarta	3,122,268	1	2	1	0.961
East Java	34,783,640	3	3	3	0.172
Bali	3,151,162	1	1	-	0.317
West Nusatenggara	4,009,261	-	-	-	-
East Nusatenggara	3,952,279	-	-	-	-
West Kalimantan	4,034,198	-	-	-	-
Central Kalimantan	1,857,000	-	-	-	-
South Kalimantan	2,985,240	-	-	-	-
East Kalimantan	2,455,120	1	1	-	0.407
North Sulawesi	2,012,098	1	-	1	0.497
Central Sulawesi	2,218,435	-	-	-	-
South Sulawesi	8,059,627	1	-	1	0.124
Southeast Sulawesi	1,821,284	-	-	-	-
Gorontalo	835,044	-	-	-	-
Maluku	1,205,539	-	-	-	-
North Maluku	785,059	-	-	-	-
Papua	2,220,934	-	-	-	-
**Overall**	**206,264,595**	**22**	**18**	**17**	**0.170**

## CONCLUSION

Much has been achieved in the 80-year history of radiotherapy in Indonesia, and the achievements in the last four years have been quite exceptional. However, shortages in many aspects of radiotherapy services serve as a reminder to the society that a lot of improvements still need to be done in various fields.

The authors also realise the limitations of the current survey, in that no measure of workload of treatment units, physicians and RTT was available. In addition, the lack of accurate data from a systematic cancer registry presents a problem in evaluating the actual need for radiotherapy. Future surveys will incorporate items from the recent EORTC recommendation [11] as well as some quality indicators [12].
